# Can video game dynamics identify orthopaedic surgery residents who will succeed in training?

**DOI:** 10.5116/ijme.58e3.c236

**Published:** 2017-04-13

**Authors:** Kenneth A. Egol, Ran Schwarzkopf, John Funge, Jeremy Gray, Christopher Chabris, Thomas E. Jerde, Eric J. Strauss

**Affiliations:** 1 NYU Hospital for Joint Diseases, NYU Langone Medical Center, New York, NY, USA. Both first authors contributed equally to the work; 2 Knack.it Corporation, CA, USA; 3 Department of Psychology, Michigan State University, MI, USA; 4 Department of Psychology, Union College, Schenectady, NY, USA

**Keywords:** Video game, dynamic, identify, orthopaedic surgery residents, training, USA

## Introduction

For those involved in orthopaedic surgery post-graduate education, it is imperative to identify and select the highest quality candidates to fill their programs.  One of the most important considerations is selecting residents who will achieve clinical competence and pass the American Board of Orthopaedic Surgeons (ABOS) certifying examination. Many candidates are also selected for their presumed future ability to contribute to the field through leadership, research, and education. Several studies have attempted to identify potential resident quality in an effort to maximize success during residency.^1^^-^^4^ Pre-residency academic achievements have been shown to correlate with higher Orthopaedic In-Training Exam (OITE) scores during residency, and well-rounded applicants (history of charity work and varsity sport participation) scored highly on faculty clinical evaluations.^3^ It has also been reported that candidates with higher United States Medical Licensing examination (USMLE) scores performed better on their board exams.^4^

In an editorial, it was remarked that the Orthopaedic Residency Review Committee’s expanded role within the Accreditation Council for Graduate Medical Education (ACGME) has enabled the orthopaedic education community to attain a number of important goals in postgraduate education with respect to interviewing and selecting candidates for residency.^5^ Therefore, it is important for orthopaedic surgery programs to evaluate and update the means by which they identify and select the candidates who are most likely to succeed during residency, fellowship, and practice.  Furthermore, identifying predictors or factors associated with success during residency would represent critical knowledge for program directors, selection committees, and applicants.

Most orthopaedic residency programs currently conduct residency selection based on faculty committee decisions. This process is commonly based on applicant interviews and CV review followed by a consensus ranking of the applicants. Such decisions are subjective and specific to each institution. To date, no substantial innovation has been introduced into the process of residency selection.

Advances in personality assessment now permit the identification of a wide range of skills, abilities, and dispositions that correlate with job success, including intelligence, conscientiousness, and social skills.^6^ Unfortunately, traditional measures of these constructs are time-consuming (sometimes costly and labor intensive) to administer, prone to self-reporting biases, and can potentially introduce error due to test anxiety and/or stereotype threat.^7^^,^^8^ An emerging trend in human resources, is the use of predictive tools to differentiate among employees or potential employees on the basis of subtle cues (not readily observable by a person) identified through data mining and machine learning.^9^

With the advent of near-universal availability of video game technology via the platforms of smart phones and other mobile devices, it has become possible to apply the same general approach to residency selection in an effort to predict job performance based on the nuances of how applicants play specifically-designed video games.^10^ Details of game-play can be used to generate statistically valid models of job performance.

The purpose of this perspective is to describe our experience in determining whether a video game-based approach is capable of predicting success in residency training.  We evaluated if a specific behavioral-trait signature could be identified that would reliably predict successful performance in an orthopaedic training program.

### Our Experience

Hundred and twenty consecutive current and former orthopaedic trainees from a single residency program were asked to play an online video game designed to measure cognitive abilities, social skills, and personality traits. 

Residency performance data was obtained from their training portfolios, namely: 1) Performance on STEP-1 of the USMLE, 2) yearly OITE scores, 3) passage of Part-I of the ABOS certifying examination, and 4) clinical skills ratings as compiled from rotation evaluations during their training. In order to be included each participant had to complete the Wasabi Waiter Video game.

### The Game

The nuances of an individual’s game-play can predict high-level psychological constructs and real-world outcomes. For example, small but reliable differences in reaction time are easily assessed from how individuals interact with the game, and how they do so in response to different kinds of game events, including the facial expressions of characters on-screen. A participant’s processing speed, accuracy, context-dependent choices, improvement over time, and other psychological features are the building blocks of subsequent analyses. The score achieved in the game is computed, but not all important by itself. What matters far more is the details of how a person engages with the game. The software measures many different things at once, making it hard for people to deliberately try to produce a desired score on a given trait.

A predictive validity was identified for those who scored higher on the OITE. Multiple game-based features (heuristics, flexibility, social perception, attentiveness, processing speed, memory, adaptive and context dependent processing speed and adaptive flexibility) contributed to the prediction of better performance on the OITE exam. A predictive validity for higher performers on the OITE combined with all clinical skills was also obtained. This model significantly predicted resident success.

The ability to identify and select people who will be successful at a given task is the goal of every human resources department. Despite having a large sample of intelligent, highly motivated applicants to choose from, orthopaedic residency programs still encounter trainees whose performance is considered subpar. In addition to assessing a trainees’ medical knowledge and clinical skills, the current training core competency structure includes several domains inclusive of behavioral characteristics. These include communication skills and professionalism. Using an online video game designed to identify players’ personality characteristics and work habits, we were able to map profiles that correlated with our residents’ performances on their training exams and their overall clinical skills.

What matters are the nuances of how people engage with the video game; a single summary score is rarely of interest. We note that this is exactly the reverse of traditional tests, for which the single summary score is all that matters. As an example, in a given game, there might be two very effective strategies for achieving a high score, e.g., one strategy that is risk-averse, and another that is risk seeking. If both strategies are effective for a given game, it means that adopting a given strategy would be unrelated to one’s overall score; however, one’s risk preference (and other traits of interest) can be inferred from the nuances of game-play and used to estimate aptitude for a profession or role for which traits like risk preference are important. Well-designed video games can have enough flexibility and richness to allow assessment of a large number of traits and dispositions simultaneously within a relatively short session.  In a previous report the authors used a personality NEO-Five Factor Inventory (FFI) to identify specific personality traits in a cohort of surgeon trainees and medical students and compared performance in a simulated operating room.^11^ They compared the personality traits of the cohort to a sample from the general population and to each other based upon performance on a laparoscopic simulator. Compared to the general population, the cohort showed less neuroticism, more extroversion and conscientiousness, and male residents showed greater openness. Despite these differences from the general population, none of the various personality styles were found to be associated with performance on the virtual reality simulator (technical performance).^11^ Thus, personality inventories may play a more important role in the non-technical aspects of residency training such as team performance.

The question as to whether certain personality types are attracted to certain medical specialties has been investigated as well. Researchers have looked at the Five Factor Model (FFM) in a group of medical students and compared them to normative data of 75,000-subjects.^12^ Surgery residents scored higher on the conscientious, extroversion and emotional stability subscales than other specialties.  With the high cost of training and the fact that certain medical specialties have been plagued with high attrition rates, 17-26%, it is critical to identify those whose personalities may be well suited for specific medical subspecialties.^12^

Burnout is another area that those in medical education have been dealing with for years.^13^ Of those who reported feeling burned out, residents were more likely to have a disorganized personality, receive less feedback about their performance, and have more uncertainty about their future career choices.^13^ Thus, identifying personality traits that could be associated with dissatisfaction in training would be helpful to allow for early intervention.

## Conclusions

Innovative video game dynamics that measure a range of behavioral markers, can help identify a profile of characteristics that may predict an orthopaedic resident’s success during training. In the future, it may be possible to predict an applicant’s potential performance during training based upon a 15-minute, video game, thus adding another important tool to the selection process of the orthopaedic surgery training programs.

### Conflicts of Interest

RS**, **KE**, **JF, JG, CC, TJ have received either fees or have stock/warrants; ES has no conflicts of interest.
